# Complex Multi-site Stereotactic Body Re-irradiation With CT-Guided Online Adaptive Radiotherapy

**DOI:** 10.7759/cureus.68559

**Published:** 2024-09-03

**Authors:** Domenic Sievert, Alden D'Souza, Xiaodong Zhao, Michael T Prusator, Tom Mazur, Hyun Kim, Dean Hobbis

**Affiliations:** 1 Department of Radiation Oncology, Washington University School of Medicine in St. Louis, St. Louis, USA

**Keywords:** hepatocellular carcinoma, sbrt, ethos, hypersight, cbct

## Abstract

Online adaptive radiotherapy optimizes a patient's treatment plan to their daily anatomy to account for inter-fraction motion. Daily target and organ-at-risk (OAR) delineation allows for optimized treatments and has been shown to have favorable outcomes in the abdominal region. Adaptive radiotherapy also has the potential to support fine control of dose in re-irradiation to OARs. Herein, we describe a complex multi-site re-irradiation case utilizing CT-guided adaptive radiotherapy. A 46-year-old man with metastatic hepatocellular carcinoma presented for re-irradiation of four metastatic lesions to the right acetabulum, T11, S2, and a gastrosplenic lymph node (gsLN). The right acetabulum, T11, and S2 lesions previously received 20 Gy in five fractions. For the current course, he was prescribed 35 Gy (T11, right acetabulum, and gsLN) and 30 Gy (S2) in five fractions. An equivalent dose in 2 Gy fractions (EQD_2_) was employed to assess cumulative doses for critical OARs and guide planning. The re-irradiated lesions were treated with stereotactic body radiation therapy (SBRT), and the gsLN was treated with adaptive radiotherapy. An isotoxic approach was utilized to create the scheduled and adapted plans for the gsLN. Adapted plans were created on the patient’s daily anatomy as visualized on kilovoltage cone beam computed tomography and compared against the scheduled plan. Dose-volume histogram objectives were used to compare the plans, and the superior plan was chosen for delivery. The adapted plan was used for all five fractions and met all critical OAR constraints while maintaining target coverage. The use of the scheduled plan would have resulted in stomach and/or esophagus constraint violations on all five fractions. This resulted in reduced EQD_2_ doses of 6.4 and 12.3 Gy for the esophagus and stomach, respectively. We report the successful treatment of a patient undergoing tri-site SBRT re-irradiation with concurrent CT-guided adaptive radiotherapy to a gsLN. The adaptive treatment allowed us to meet critical OAR constraints while maintaining target coverage. Few studies have described the use of CT-guided adaptive radiotherapy in re-irradiation cases, and the potential benefit for these complex cases is evident.

## Introduction

Hepatocellular carcinoma (HCC) is the most common malignant liver tumor and is the third leading cause of cancer-related deaths worldwide and the seventh in the USA [1,2]. In the US, HCC has had a rising incidence and mortality over the last two decades [1]. The most frequent etiology is cirrhosis, with common risk factors including viral hepatitis, alcohol, and metabolic dysfunction-associated liver disease [2,3].

Treatment for HCC has various modalities, including but not limited to liver transplant, radiofrequency ablation, microwave ablation, transarterial chemoembolization or radioembolization, targeted therapy, immunotherapy, or radiotherapy. Traditionally, about 20% of patients with HCC were eligible for the primary curative options of surgery and liver transplant, and external beam radiation therapy was generally delivered within a palliative context. Over the last decade, stereotactic body radiation therapy (SBRT) has been emerging as an option for locoregional control [4,5]. SBRT is starting to be adopted with curative intent for oligometastatic HCC [6]. Additionally, this is further supported by an ability to achieve ablative doses in adaptive SBRT for non-bone oligometastases [7]. Herein, we describe the application of CT-guided adaptive therapy combined with non-adaptive SBRT for multi-site re-irradiation therapy.

## Case presentation

Patient presentation

**Figure 1:**
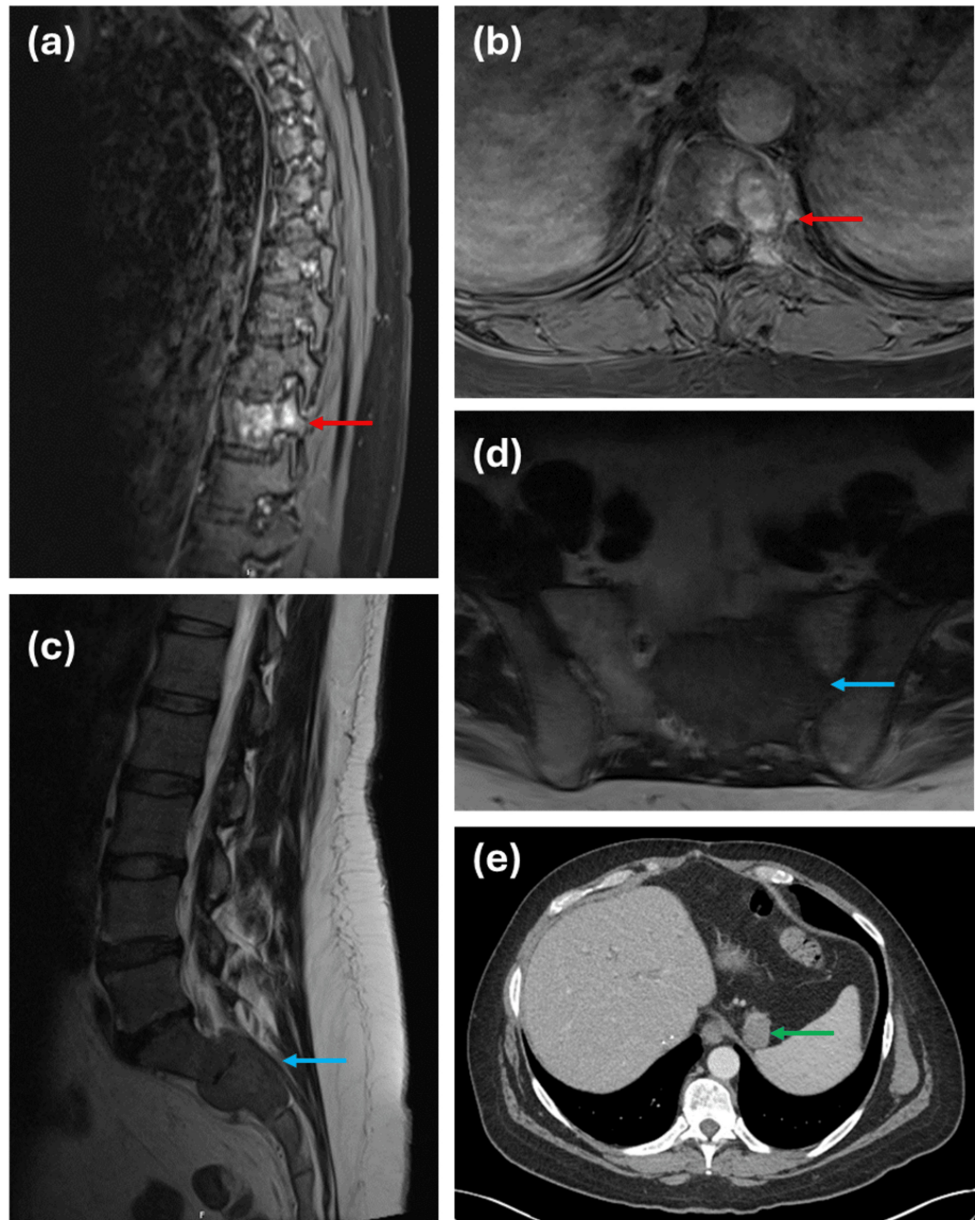
The identified lesions for (a-b) T11 and (c-d) S2, as shown on MRI, and (e) gastrosplenic lymph node, as shown on CT.

A 43-year-old man was diagnosed with HCC secondary to alcoholic cirrhosis while being assessed for a liver transplant. He then had transarterial chemoembolization as a bridging treatment to a liver transplant that month. Three years later, he presented with left-lower extremity numbness and fecal incontinence, which improved with steroids and oral analgesics. His diagnostic MRI showed a 3.4 × 1.3 × 2.0 cm mass at T11, a 6.1 × 3.6 × 4.6 cm expansile mass of the S2 extending to the S1 vertebral body, and his diagnostic CT showed a 2.0 cm gastrosplenic lymph node (gsLN) lesion (Figure 1). The patient had a sacral biopsy that was consistent with metastatic HCC. He was seen by the medical oncology team who created a plan for systemic therapy with lenvatinib and planned for an outpatient referral to radiation oncology.

Radiation oncology created a primary plan for definitive intent SBRT to his oligometastatic disease. A fluorodeoxyglucose-PET/CT scan showed an additional metastasis to his right acetabulum and confirmed the three above lesions. Insurance denial for SBRT to all four sites led to initial treatment of 20 Gy in five fractions to the T11, S2, and right acetabular sites. Follow-up CT imaging at two months post-treatment demonstrated progression at the four aforementioned sites, thus his systemic therapy was adjusted, and the patient was offered SBRT to all four sites. His gsLN was treated with adaptive SBRT, while the acetabulum, T11, and S2 lesions were treated without adaptation.

Treatment planning and delivery

Pre-treatment Imaging

The patient was immobilized for CT simulation in a custom immobilization device with both arms overhead. The scan extent included all four lesions, extending from the base of the skull to below the inferior boundary of the iliac crest. Acquired scans included a fast helical CT, retrospectively phase-binned four-dimensional CT (4DCT), and end-exhale breath-hold CT with IV contrast administered with a 45-second delay. As no contrast was administered during treatment, the contrast was overridden to the density of water for treatment planning purposes. The 4DCT indicated that the gsLN overlapped with the T11 spine lesion in the inhale phase. Treatment planning was performed on the end-exhale breath-hold and free-breathing CTs for the gsLN and other sites, respectively.

To ensure patient compliance with breath-hold and suitability for adaptation, patient setup was repeated on the ETHOS treatment platform (Varian Medical Systems, Palo Alto, CA) immediately following CT imaging, and a cone-beam computed tomography (CBCT) was acquired to assess anatomy visualization. T2-weighted (T2W) and T1-weighted (with and without gadolinium contrast) MRIs were acquired and independently co-registered with simulation CT datasets to aid in delineating the target volumes and spinal cord. The gsLN was planned in the ETHOS (v1.1.0) treatment planning system (TPS). The three non-adaptive SBRT sites were planned with separate isocenters in the Eclipse (v15.6.06) TPS (Varian Medical Systems) and delivered on a Varian EDGE linear accelerator (Varian Medical Systems) with high‐definition multileaf collimators. The gsLN gross tumor volume (GTV_gs_) was delineated based on co-registered MRI and expanded by 5 mm to create its planning target volume (PTV_gs_).

Similarly, the right acetabulum gross tumor volume (GTV_r_acet_) and sacrum gross tumor volume (GTV_sacrum_) were contoured, as visible on the simulation imaging, with a 0.5 cm expansion for planning target volume (PTV) definition. The T11 spine SBRT lesion clinical target volume (CTV_T11_) was contoured using international consensus guidelines and included the entire vertebral body and the ipsilateral (left) pedicle/transverse process [8]. The PTV_T11_ was generated by a 0.2 cm isotropic expansion of the CTV_T11_. The radiation oncologist also specified the spinal cord at the time of target delineation, as defined by T2W MRI, and extending at least one vertebral body above and below the involved vertebrae.

Critical OAR Considerations - EQD_2_

The T11, right acetabulum, and sacrum SBRT sites were treated after previous treatments to 20 Gy in five fractions four months prior to the current course. Additionally, the 4DCT dataset indicated an overlap in the axial plane of the gsLN and the T11 target, requiring special considerations during planning. Critical organs at risk (OARs) assessed using a composite dose distribution with current and prior courses included the spinal cord, stomach, esophagus, duodenum, large bowel, small bowel, rectum, and sacral plexus. Cumulative dose constraints were specified as equivalent dose in 2 Gy fractions (EQD_2_) [9]. The constraint for the spinal canal was specified conservatively relative to the Hypofractionation Treatment Effects in the Clinic (HyTEC) recommendation as EQD_2_ D0.03cc < 65 Gy (α/β = 2) given that prior treatment was less than five months prior [10]. The esophagus EQD_2_ constraint was D0.5cc < 75 Gy and the stomach constraint was D0.5cc < 98 Gy (α/β = 3) [11,12]. Cumulative dose constraints were evaluated by taking the maximum doses to the specified volume from each individual plan. Thus, this approach is considered conservative as it does not account for spatial variation in dose volumes between the plans.

Adaptive Planning

For the gsLN, a PTV_gs_ optimization (PTV_gs_opt_) structure was derived from PTV_gs_ by subtracting overlap with heart, stomach, and esophagus plus a margin, more specifically ((PTV - (heart + 0.1 cm)) - ((stomach + esophagus) + 0.3 cm)). Optimization was prepared to push the prescription dose to PTV_gs_opt_ and a minimum dose of 25 Gy to PTV_gs_ while ensuring the highest priority OAR constraints are met for each fraction [13-15]. Two coplanar arcs about the patient's left side (gantry angles: 345.0-179.9 degrees) were used with collimator angles of 15 and 345 degrees. Daily re-contouring was confined to an anisotropic contour ring contour generated from the PTV_gs_ (S/I: 1.2 cm, L/R/A/P: 3 cm). Scheduled (P_gs_S_) and adapted plans (P_gs_A_) were created based on the patient's daily CBCT to account for inter-fractional anatomy variation. A CBCT was acquired under an end-exhale breath-hold utilizing a surface tracking system with a 2 mm vertical amplitude margin [16]. The GTV_gs_ contour was rigidly propagated from the simulation CT to the CBCT and aligned. The OARs are deformed before contour editing. The covering radiation oncologist edited the OARs within the contour ring and GTV_gs_ as necessary [17]. The plan was then re-optimized on the daily anatomy to generate P_gs_A_. Then P_gs_S _and P_gs_A_ are displayed on the CBCT for comparison. The plans were evaluated using dose volume histogram (DVH) objectives. The P_gs_A_ plan is chosen if the target coverage improves over the P_gs_S_ by > 5% or if P_gs_A_ meets a hard OAR objective (priority 1 constraint) not met by P_gs_S_.

SBRT Planning

The T11 spine target was planned with two complete arcs and collimator rotations of 100 and 80 degrees for efficient spinal cord blocking. The 4DCT indicated that the motion of the inferior portion of the GTV_gs_ overlapped with the axial planes of T10 and T11. An optimization stomach avoidance structure was manually delineated as an avoid entry structure for the spine plan to prevent dose to GTV_gs_ and to help shape dose fall off in the region between the PTV_gs_ and PTV_T11_ to help meet the minimize dose to the stomach and to meet the EQD_2_ constraint. kV-triggered imaging was used to monitor intra-fractional motion during treatment. This is realized by generating an NS_Trigger_T11 structure encompassing the directly adjacent vertebral bodies (T10-T12) with an additional 2 mm margin. The sacrum plan had two complete arcs and shallow collimator angles (345- and 15-degrees). The optimization constraint of the hotspot for the sacrum plan was 105% to spare the sacral plexus overlapping target. The right acetabulum plan was designed using two half arcs about the patient's right side with 350- and 10-degree collimator angles.

Dosimetric and clinical results

**Figure 2:**
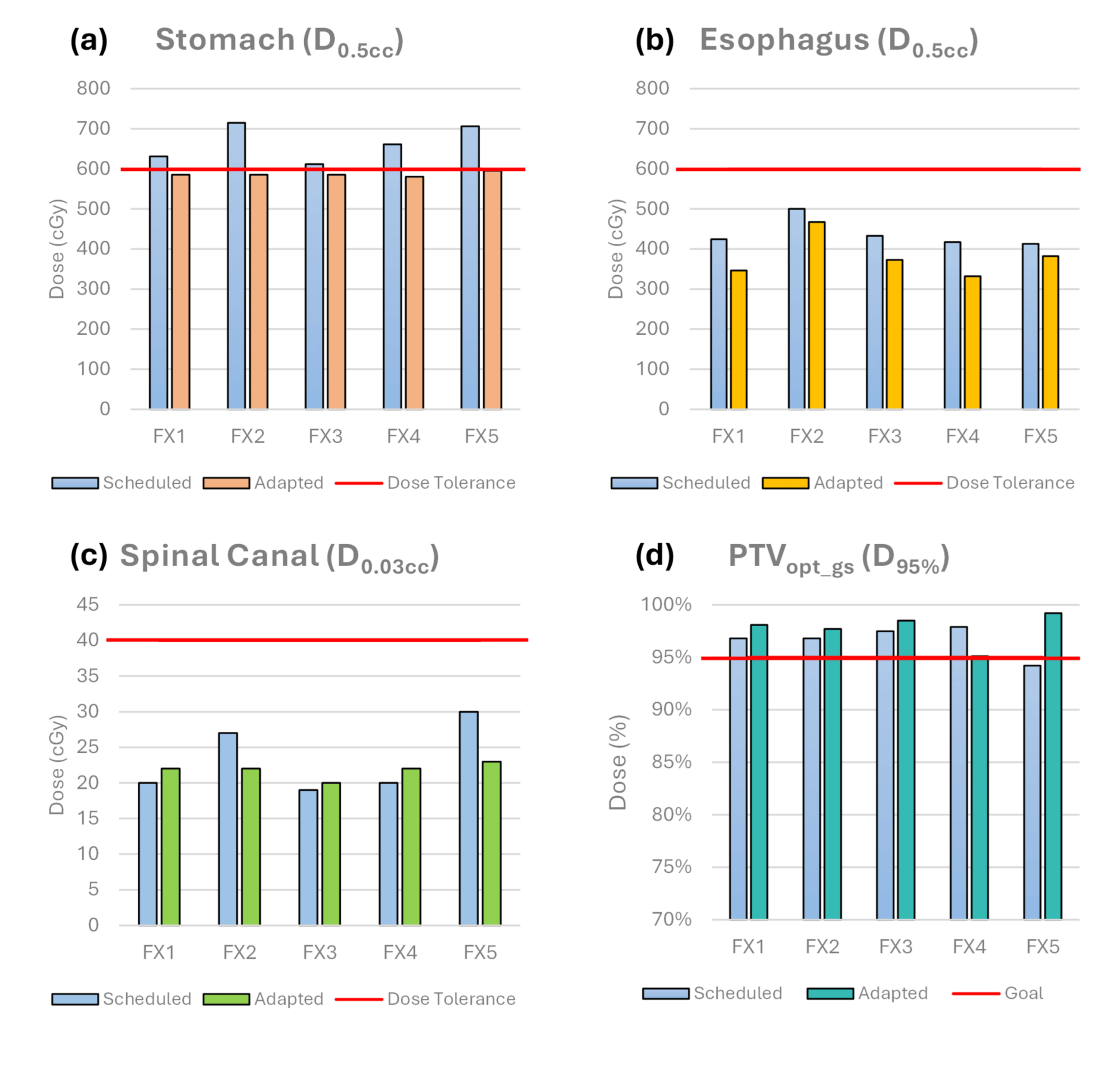
Physical doses per fraction for the scheduled and adapted plans for (a) stomach, (b) esophagus, (c) spinal canal OAR constraints, and (d) optimization target volume coverage. D_x.xxcc_: dose to x.xx cubic centimeters; D_xx%_: dose to xx% of the total volume; OAR: organ at risk; PTV: planning target volume.

**Figure 3:**
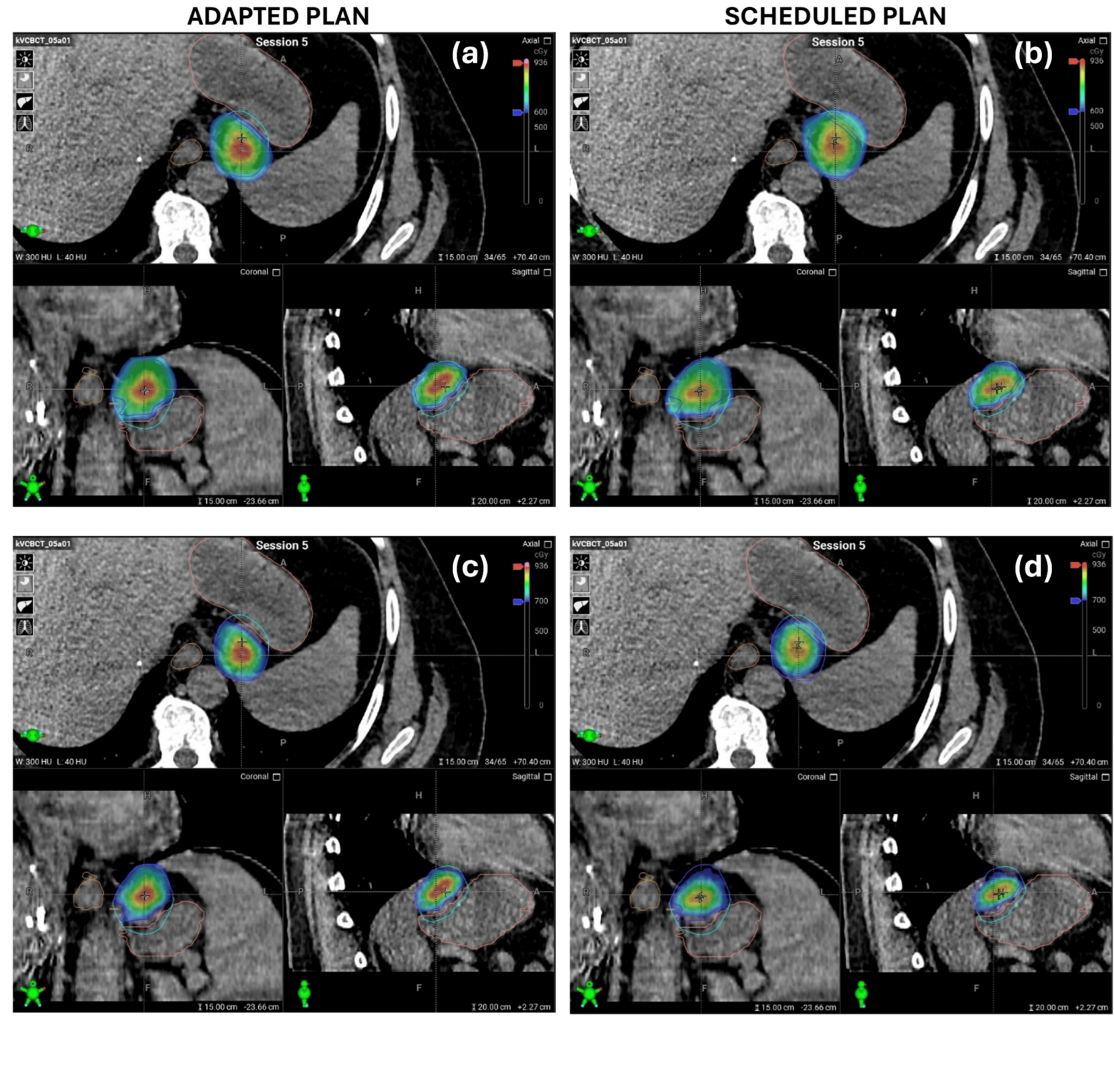
Comparison of the adapted versus scheduled plan for fraction five of the adapted gastrosplenic lymph node treatment. The 600 cGy isodose line represents the fractional OAR dose constraint and is shown for the (a) adapted and (b) scheduled plan, respectively. The 700 cGy isodose line represents the goal for optimization of PTV coverage and is shown for the adapted (c) and scheduled (d) plans. OAR: organ at risk; PTV: planning target volume.

The average total treatment time for all five adaptive fractions was 61.1 minutes (range: 54.7-70.7 minutes) (Table 1). The scheduled plan did not meet the priority 1 stomach constraint on any fractions and was the primary reason for adaptation, as seen in Figure 2. PTV_gs_opt_ received greater than 95% of the prescription dose in all adapted fractions, with an average increase of 1.1% over that of the P_gs_S_. The median (IQR) dose for GTV D_98%_, PTV D_95%_, and PTV_gs_opt_ D_95%_ were 30.6 (1.0), 29.5 (0.0), and 33.8 (2.5) Gy for P_gs_S_, and 30.0 (1.2), 29.1 (0.8), and 34.2 (0.3) Gy for P_gs_A_, respectively. Figure 3 shows a qualitative comparison of the scheduled versus adaptive plans for fraction 5. The stomach would have received 6 Gy to 5.58 cc for this fraction if treated with the scheduled plan, compared to only 0.36 cc for the adaptive plan. Moreover, PTV_gs_opt_ D_95%_ coverage increased by 5.0%. EQD_2_ comparisons for the stomach, esophagus, and spinal canal are compared in Table 2. The spinal canal EQD_2_ did not decrease for the adaptive approach; however, the esophagus and stomach saw reductions of 6.4 and 12.3 Gy, respectively. The multiple sites' rectum and other luminal GI structures met their EQD_2_ constraints.

**Table 1:** Treatment times for each adaptive fraction (from the initial image to the patient off the table).

Fraction #	1	2	3	4	5
Time (m:s)	70:44	57:28	54:40	65:28	57:03

**Table 2:** EQD2 values in Gy for the critical OARs for the adaptive abdominal lymph node radiotherapy course with previous treatments. Comparison of the values if treated with the scheduled and adapted plans as calculated on the daily anatomy. EQD_2_: equivalent dose in 2 Gy fractions; D_x.xxcc_: dose to x.xx cubic centimeters; SBRT: stereotactic body radiation therapy; 3D: three-dimensional; gsLN: gastrosplenic lymph node; P_gs_S_: scheduled gastrosplenic lymph node plan; P_gs_A_: adapted gastrosplenic lymph node plan; OAR: organ at risk.

Treatment plan	Stomach - D_0.5cc_ (Gy)		Esophagus - D_0.5cc_ (Gy)		Spinal canal - D_0.03cc_ (Gy)	
α/β	3		3		2	
T11_3D_	18.3		13.8		31.6	
T11_SBRT_	6.3		1.5		26.7	
gsLN_SBRT_	P_gs_S_	P_gs_A_	P_gs_S_	P_gs_A_	P_gs_S_	P_gs_A_
	64.2	51.9	32.3	25.8	0.7	0.6
Total	88.8	76.5	47.5	41.1	59.0	58.9

## Discussion

We describe in this report a unique and complex SBRT re-irradiation case using CT-guided online adaptive radiotherapy. Compared to conventional radiotherapy, SBRT is associated with higher precision, smaller target margins, and steeper dose gradients. These characteristics may make SBRT more advantageous for re-irradiation cases. The efficacy and safety of spine SBRT are well characterized [18], whereas non-adaptive image-guided abdominal SBRT is less established. Recent studies have shown that adaptive abdominal SBRT is associated with improved clinical and dosimetric outcomes [19]. Adaptive SBRT can provide additional benefits for re-irradiation cases with the potential to tailor dose on daily anatomy to optimally cover the target volume while minimizing dose to critical OARs, with the potential to be able to reduce margins further. The inter- and intra-fractional motion of the structures in the abdominal region makes re-irradiation in this region a prime candidate for adaptive SBRT. For the presented case, the ability to deliver P_gs_A_ prevented violation of the stomach's hard constraints on all five fractions while maintaining comparable target coverage between P_gs_S_ and P_gs_A_. Fraction 5 (Figure 2D) shows the greatest increase in PTV_opt_gs_ coverage, partly because GTV_gs_ was edited on treatment. Similarly, GTV_gs _was edited on fraction 1, with the rigid alignment of GTV_gs_ sufficient for the remaining three fractions. Identifying critical OAR goals prior to the treatment planning process is paramount to the efficient use of resources and plan quality for reirradiation cases. However, consensus data for dose tolerances in the re-irradiation setting are not well defined and a conservative approach should be taken in such scenarios. During the planning process, a continuous line of communication was opened between the physician, dosimetry, and physics teams for a dynamic approach to optimizing and refining the individual treatment plans to achieve the cumulative dosimetric goals.

**Figure 4:**
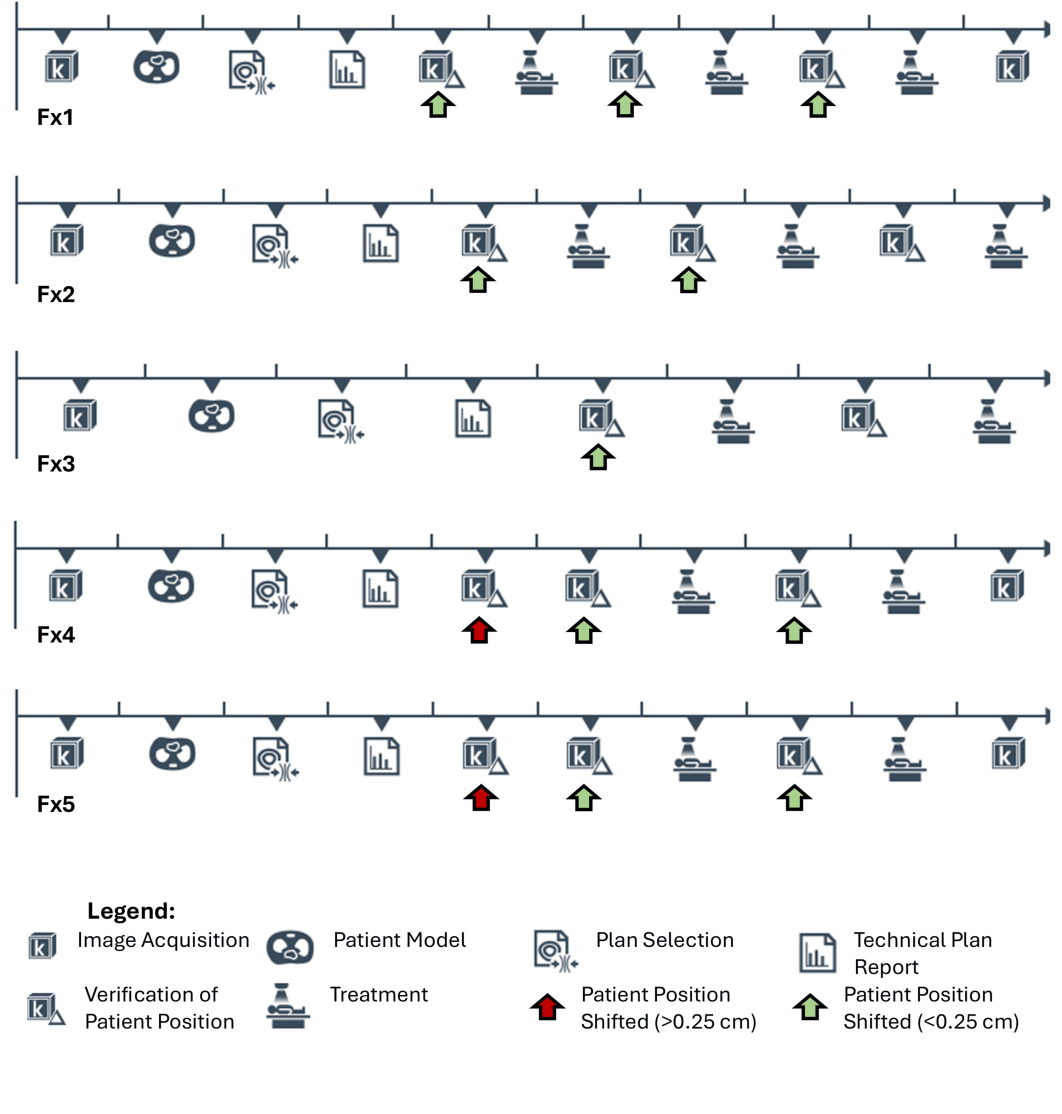
Step-by-step timeline for each treatment fraction (from initial image to patient off the table). Image created using screenshots from the ETHOS treatment planning system monitor workspace.

Efficient treatment delivery is critical when patients have multi-site treatments across multiple treatment machines. This reduces the strain on the patient and the radiation oncology team, where patient compliance is paramount, particularly for adaptive treatments that require long times on the treatment table and are more resource-intensive. On the first fraction, patients must re-learn breathing instructions, physicians and physicists must acquaint themselves with the variation in anatomy from the CT simulation, and therapists must set up the surface- and image-guided protocols. These factors are attributed to the longer time reported for the first fraction, which is then reduced for the subsequent treatments. The challenge with re-learning breathing instructions is highlighted in Figure 4, where the treatment beams were interrupted by the need to acquire a new verification CBCT in the first two fractions due to baseline drift in the optical surface monitoring of the patient’s breathing trace. Beyond the second fraction, the patient's breathing became more consistent and did not drift from baseline. The verification CBCT acquired directly prior to delivery of the adapted plan indicated the need to shift the patient on each fraction. This is due to intra-fractional anatomy changes while the adapted plan is generated. Similar shifts were required at the mid-treatment verification CBCTs in four of the five fractions. At our institution, if the CBCT shifts are <0.25 cm (0.5*PTV_margin_), then a repeat (or back-to-back) CBCT is taken before beam delivery. This can be seen for fractions 4 and 5 at the pre-treatment verification CBCT stage.

## Conclusions

Herein, we demonstrate the dosimetric benefit of CT-guided adaptive SBRT as a component of a highly complex multi-site re-irradiation case. Adaptive CT-guided SBRT has the potential for improved dosimetry for SBRT re-irradiation cases. The ability to adapt each day may greatly benefit re-irradiation patients as the prioritization of OAR dose constraints and target coverage goals can be dynamically evaluated during treatment, ensuring a more optimal balance. Although clinical trials are needed to investigate the impact on toxicity and local control.
